# Resource Allocation for Machine-Type Communication of Energy-Harvesting Devices in Wi-Fi HaLow Networks

**DOI:** 10.3390/s20092449

**Published:** 2020-04-25

**Authors:** Dmitry Bankov, Evgeny Khorov, Andrey Lyakhov, Jeroen Famaey

**Affiliations:** 1 Institute for Information Transmission Problems, Russian Academy of Sciences, 127051 Moscow, Russia; bankov@iitp.ru (D.B.); lyakhov@iitp.ru (A.L.); 2 Telecommunication Systems Lab, National Research University Higher School of Economics, 101000 Moscow, Russia; 3 IDLab, Department of Computer Science, University of Antwerp-imec, 2000 Antwerp, Belgium; jeroen.famaey@uantwerpen.be

**Keywords:** machine-to-machine communications, energy harvesting, Internet of Things, Wi-Fi HaLow, IEEE 802.11ah

## Abstract

The recent Wi-Fi HaLow technology focuses on adopting Wi-Fi for the needs of the Internet of Things. A key feature of Wi-Fi HaLow is the Restricted Access Window (RAW) mechanism that allows an access point to divide the sensors into groups and to assign each group to an exclusively reserved time interval where only the stations of a particular group can transmit. In this work, we study how to optimally configure RAW in a scenario with a high number of energy harvesting sensor devices. For such a scenario, we consider a problem of device grouping and develop a model of data transmission, which takes into account the peculiarities of channel access and the fact that the devices can run out of energy within the allocated intervals. We show how to use the developed model in order to determine the optimal duration of RAW intervals and the optimal number of groups that provide the required probability of data delivery and minimize the amount of consumed channel resources. The numerical results show that the optimal RAW configuration can reduce the amount of consumed channel resources by almost 50%.

## 1. Introduction

The concept of the Internet of Things (IoT) [1] with its tremendous number of interconnected autonomous devices has become very attractive for device vendors, mobile operators, and their customers [2]. In addition to whipping up the telecommunication market, IoT brings new services and applications, which will revolutionize our life. From the technical point of view, it is evident that the easiest way to provide Internet access for the swarm of devices is using wireless communications. At the same time, it is not clear how to do it the most efficiently.

Another challenge is the energy consumption. Since it may be difficult, if even possible, to wire thousands of devices to an electric grid, autonomous devices are typically battery-supplied and/or harvest solar, wind, or other kinds of green energy. Using green energy may be especially fruitful in rural deployments, e.g., for agriculture monitoring. However, energy harvesting still needs energy storage. Unfortunately, from both technical and ecological points of view, the usage of traditional accumulators by autonomous devices is limited. First, their efficiency degrades with every recharge cycle. Second, they typically contain much mercury, cadmium, lead, or other dangerous poisonous materials. That is why, in many scenarios, autonomous devices have rather small energy accumulators or even capacitors [3,4,5].

Both low energy consumption and a high number of devices occasionally transmitting short messages are the main challenges for IoT networking protocol developers. To handle them, many international organizations and standardization bodies are currently developing new telecommunication standards or adapt existing communication technologies to IoT. Thus, LoRa can be used together with vibrational energy harvesters for bridge monitoring [6] or with RF energy harvesters for health monitoring in construction [7]. Another example of the successful application of telecommunication technologies with energy harvesting is the usage of Bluetooth Low Energy (BLE) with ambient light energy harvester [8] or RF energy harvesters [9] in Smart Building scenarios, e.g., to track the room occupancy.

LoRa and BLE have been designed for long and short range communications, respectively, and provide rather low data rates. The intermediate niche can be occupied by the Wi-Fi Halow technology. It is based on the IEEE 802.11ah [10] amendment to the Wi-Fi standard, and is designed to make Wi-Fi suitable for communication of swarms of autonomous devices [11]. Similarly to LoRa, it operates in sub 1 GHz frequencies, but uses wider channels (from 1 MHz to 16 MHz) and can provide much higher data rates (up to ≈347 Mbps). As a result, it can be used in sensing scenarios, such as residential scenarios (smart meters, elderly care) or environmental and agricultural monitoring with a transmission range of < 1km [11]. Such scenarios are a perfect match for energy harvesting batteryless sensors [8,9,12] to be used in conjunction with Wi-Fi HaLow. These sensors can harvest RF, thermal, solar, or mechanical energy [13].

A key component of the 802.11ah amendment is the Restricted Access Window (RAW): a novel channel access method, which allows for reducing the number of stations (STAs) accessing the channel simultaneously and thus limits the contention for channel access between the STAs. The contention of a high number of STAs results in collisions. According to [14], collisions are the main source of energy losses in Wi-Fi networks.

RAW allows an Access Point (AP) to divide the STAs into several groups and assign each group to a time interval during which only this group can access the channel. Thus, RAW allows the AP to limit contention between energy limited STAs. In addition, it protects the transmission of these STAs from collisions with other STAs, e.g., offloading STAs connected to the same AP.

Offloading is the second important use case for IEEE 802.11ah thanks to the long transmission range and relatively high maximal throughput. It is a feasible and cheap way to meet the clients’ requirements on throughput and latency in cellular networks. In a big city, cell phone users are in Wi-Fi zones for 70% of their time, and 65% of mobile traffic can be offloaded [15]. An IEEE 802.11ah network can be used both for gathering data from energy-limited sensors and for serving the offloading STAs, and there is a trade-off between the channel resources allocated to each kind of STA. On the one hand, enough channel resources should be allocated (with RAW) to the energy-limited STAs so that they can deliver their data. On the other hand, it is necessary to leave as many channel resources to the offloading STAs as possible to avoid network underutilization.

The RAW mechanism has been extensively studied via simulation [16,17] and mathematical modeling [18,19,20,21,22,23,24,25,26,27,28,29]. In this paper, we focus on analytical approaches to estimate the network performance and note that most papers that analyze the efficiency of Wi-Fi networks with RAW incorrectly describe the data transmission with RAW. Most related works [18,19,20] consider saturated traffic which is not a typical IoT scenario, while those that consider non-saturated traffic [21,22,23,24,25,26] mostly neglect the fact that the data transmission process within the allocated time intervals is non-stationary, i.e., the probabilities of transmission attempt and collision change with time. At the same time, the papers which correctly model the non-stationary data transmission process [27,28,29] do not consider the possibility of STAs running out of energy during the transmission process, but such an event is quite likely and important in IoT scenarios due to the following reasons. Firstly, the packet losses in wireless sensor networks can be caused not only by noise in the channel and contention for channel access but also by the depletion of STA energy. Secondly, when STAs run out of energy, they stop contention for channel access, and thus, the conditions for the other STAs are improved, which also affects the packet loss rate. Thus, it is important to consider the amount of energy available for the STAs while studying the data transmission solutions for wireless sensor networks.

The core contribution of this paper is the performance evaluation of the channel access method introduced in the 802.11ah amendment in networks with energy-harvesting devices. For that, we significantly extend our approach previously developed in [27,28] to model RAW behavior. In contrast to [27,28], in this paper, we consider a more complex scenario when we develop our mathematical model.

First, the STAs are energy harvesting and are supplied by a limited source of energy, so, during the transmission within the RAW slot, an STA can stop contending for the channel because of the depletion of the energy. This factor is important for two reasons. On the one hand, an STA that does not have enough energy fails to deliver its traffic during the RAW slot. On the other hand, when other STAs stop contending for the channel, the probability of successful transmission for the considered STA increases.

Second, the transmission attempts can fail because of collisions and the random noise in the channel, which is new.

With the developed mathematical model and simulation, we find non-obvious effects, which we describe and explain in the paper. Finally, we show how to use the model to find an optimal RAW configuration, i.e., such RAW parameters that minimize channel time consumption by the energy-harvesting devices and, at the same time, guarantee the delivery of the data with the required probability. We also show that the usage of models that does not take into account the limited sensor’s energy and the parameters of random noise in the channel can lead to an incorrect choice of RAW parameters, which cannot provide the required probability of data delivery.

The rest of the paper is organized as follows. In Section 2, we describe the studied scenario in more detail and state the problem solved in the paper. Section 3 reviews the papers related to the subject of wireless energy-limited device networks, IEEE 802.11ah networks, and data transmission in Random Access Window. In Section 4, we design an analytical model to solve the stated problem. In Section 5, we present and discuss the numerical results obtained with the model. Section 6 summarizes the main results of the study.

## 2. Problem Statement

### 2.1. Channel Access Method

The core idea of RAW is that an AP can select several STAs and allocate them a time interval called the RAW slot, during which only the selected STAs can transmit their packets while the other STAs are forbidden to access the channel. The AP can allocate a single RAW slot to a group of STAs or can establish a long-term periodic RAW slot allocation (so-called Periodic RAW or PRAW). With PRAW, the AP allocates to a group of STAs a series of equidistant time intervals of the same duration. In this article, we consider the PRAW approach since it introduces less overhead than repeating a single RAW slot allocation.

To access the channel during its RAW slot, the STA uses a native Wi-Fi enhanced distributed channel access (EDCA), which implements Carrier Sense Multiple Access with Collision Avoidance (CSMA/CA). In summary, it works as follows. At the beginning of the slot, the STA initializes a backoff counter with a random integer value equiprobably drawn from the interval $\left[0,CW_{0}-1\right]$, where $CW_{0}$ is the minimal contention window (a configurable network parameter). If the channel is idle during time σ, the STA decrements the backoff counter. If the channel is busy, the STA freezes its backoff counter and waits until the medium becomes free. It unfreezes the backoff counter when the channel has been idle during the arbitration interframe space (AIFS). When the backoff counter reaches zero, the STA transmits its data frame, provided that the transmission, including the expected acknowledgment frame (ACK), ends before the termination of the RAW slot.

If a frame transmission fails because of the random noise or collisions, the ACK does not arrive. Thus, the STA considers the transmission attempt as unsuccessful and increments the retry counter *r* (initially set to zero). If the retry counter reaches the Retry Limit RL, the pending frame is dropped. Otherwise, the STA repeats the transmission attempt. For that, it equiprobably draws a new backoff value from doubled contention window $\left[0,CW_{r}-1\right]$, where

(1) $$CW_{r}=\min\left\{CW_{max},2CW_{r-1}\right\}.$$

### 2.2. Scenario

Let us consider a network with an AP, many energy harvesting sensor STAs, and several offloading STAs. From time to time, energy-harvesting STAs generate some small portion of information and intend to transmit a packet with this information to the AP. The packet shall be delivered to the AP no later than $D_{max}$ after it has been generated. Otherwise, it becomes outdated and is discarded by the STA. Additionally, the ratio of delivered packets shall not be less than $p_{req}$. To simplify the further analysis, we assume that all packets have the same size.

**Figure 1:**
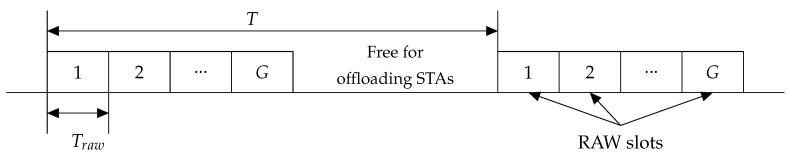
The Periodic RAW scenario.

The AP divides energy-harvesting STAs into several groups and assigns a periodic series of RAW slots to each group. The energy-harvesting STAs transmit their packets only in their own RAW slots. The offloading STAs are allowed to transmit only outside the allocated RAW intervals, as shown in Figure 1.

We consider that the STAs assigned to the same RAW slot are located in the transmission range of each other, i.e., they are not hidden from each other. Such an assumption is reasonable because IEEE 802.11ah provides several ways to avoid hidden STAs and improve performance. First, during the association process, the AP can determine the direction for each sensor STA and then group STAs with respect to their locations. Second, it can use the sectorization mechanism, which allows only those STAs to transmit that are located in a given sector [18,30].

Every STA generates a Poisson flow of packets, the intensity of which is much lower than $\frac{1}{D_{max}}$ and the period of RAW slots allocated to the STA. Having generated a packet, an energy-harvesting STA waits for its slot and tries to deliver the packet. A transmission attempt may fail because of the random noise in the channel or collisions. The STA’s transmission is damaged by noise with probability *p*.

If the transmission attempt is unsuccessful, the STA repeats it if the retry limit is not reached. As soon as the packet is successfully delivered, the STA switches off its radio interface to save energy.

The STAs are supplied by a capacitor or small accumulator and can slowly harvest the energy from the environment, e.g., using a solar cell, motion, or an RF energy harvester [31]. We assume that at the beginning of the RAW slot, the STA has a random amount *Q* of energy, which it has harvested since the last transmission attempt. When an STA listens to the channel or tries to transmit a frame, it consumes energy. As in [32,33,34], if the STA’s energy becomes less than the amount required to transmit a frame, the STA switches off its radio interface at least until the next RAW slot, even if its packet is not delivered. In addition, the STA switches off its radio at the end of the RAW slot.

Since this research is focused on the evaluation of channel access in Wi-Fi HaLow networks using RAW, we consider a simplified model of energy harvesting and, similarly to [32,34,35], assume that the amount of energy that the STA harvests since the last transmission attempt linearly depends on the elapsed time, i.e., we consider that the fluctuations related with the instability of the energy source, e.g., the variability of the solar flux caused by the clouds, are averaged over a sufficiently large period of time. In our scenario, it is justified by the assumption that the RAW period is much higher than the RAW slot duration. The average amount of 〈Q〉 of energy harvested by the STA within a RAW period is a variable parameter that characterizes the energy harvesting system, e.g., the solar cell area.

As the frame delay budget is $D_{max}$, the period of RAW slots shall not be greater than $D_{max}$. At the same time, the period of RAWs less than $D_{max}$ is also inefficient. To clarify this statement, let us consider a group of STAs that is granted a series of RAW slots. Let us fix the channel resource consumption defined as the time that the group is allowed to transmit and consider two cases. In the first case, RAW slots follow with period *T*, and their length is $T_{raw}$. In the second case, RAW slots follow with period αT and their length is $\alpha T_{raw}$, where α<1. In the latter, STAs have less time for transmission, but the more important problem is that the contention windows the STAs had at the end of the RAWs are not kept. Therefore, they spend more time with small contention windows and hence high collision probability, wasting energy. For that reason, we consider only the scenario when the RAW period is $D_{max}$.

For the described scenario, we state the problem to find out how to divide STAs into groups, and to determine the RAW slot duration for each group in such a way that the channel resource consumption by the STAs is minimized, but the restriction on minimal packet delivery ratio $p_{req}$ is met. To solve this problem, we develop a model of the described above process.

## 3. Related Papers

In the literature, many papers study various aspects of energy-efficient data transmission in wireless sensor networks. A group of studies consider abstract wireless sensor networks and derive general models of their operation using the Markov models [36], Energy Packet [37,38] abstraction, or the Brownian motion [39]. Another group of studies consider wireless sensor networks using different technologies, including the cellular networks [40,41] such as NB-IoT [42], low power wide area networks such as LoRaWAN [43,44], and wireless personal area networks such as ZigBee [45]. Finally, some papers evaluate the benefits of low-power wake-up radios added to existing technologies to reduce power consumption, e.g., see [46,47] for Wi-Fi and 5G networks. However, since this paper is focused on the peculiarities of channel access in Wi-Fi HaLow, we further review the literature specifically on this technology.

Although there are many studies of RAW and other mechanisms introduced in the IEEE 802.11ah amendment, which evaluate the efficiency of these mechanisms via simulation [16,17], we focus on the works containing analytical approaches to estimate the network performance.

Most papers that analyze the efficiency of Wi-Fi networks (e.g., [48]) consider networks operating in saturated conditions, i.e., when all STAs always have data to send, and all STAs are allowed to transmit. Many papers [18,19,20] that analyze Wi-Fi HaLow with RAW consider the same scenario, and the presented analysis is mostly based on the assumption that the network operates in stationary conditions so that the probabilities of device transmission and collision are constant. However, the usage of such an approach for analysis of Wi-Fi HaLow networks with RAW is poorly justified in the case of many Internet of Things applications due to two reasons. The first reason is that the typical IoT traffic is not saturated, i.e., STAs rarely transmit small pieces of information. The second reason is that the STAs reset their backoff functions at the beginning of the RAW slot: the STAs use the smallest contention windows at the RAW slot start and increase their contention windows with every unsuccessful transmission attempt. As a result, the probability of an STA transmitting in a given instant of time changes with time during the RAW slot, and the analysis based on stationary transmission probability is a priori inaccurate. This fact is confirmed in [29], where the authors develop a model of data transmission in RAW and compare its results with an approach that does not take into account the fact that the STAs start transmission in a RAW slot with new backoff functions, and show that such an approach can lead to a very high relative error, up to 400%.

The transmission of non-saturated data streams in Wi-Fi HaLow is considered in [21,22,23,24,25,26]. In [21], the authors propose a channel access scheme that is built upon RAW and analyze it for a case of non-saturated traffic. In [22], the authors propose a retransmission scheme that uses empty RAW slots to resolve collisions and, thus, to decrease energy consumption. However, in both [21,22], the resulting channel access is not the standard EDCA used in Wi-Fi HaLow, but ALOHA-like access. In [23], the authors describe data transmission in a RAW slot in the case when an STA can have one packet for transmission and derive the energy consumption by the STAs. In [24], the authors consider non-saturated load and non-ideal channel conditions and derive formulae for the network throughput and backoff duration. The authors also suggest leaving a portion of channel time open for access so that the STAs that cannot transmit their data during their RAW slots could finish their transmissions. This research is further extended in [25] to a case with several EDCA access categories. In [26], the authors develop a model of data transmission with RAW, find the average energy consumption of device groups, and present a traffic grouping approach that can be used to minimize the energy consumption. Although [21,22,23,24,25,26] study a scenario with non-saturated traffic, more relevant to the IoT, all these papers neglect the fact that the data transmission process within a RAW slot is not stationary, and the probabilities of collision and success change with time.

The first accurate analysis of the non-stationary data transmission process in RAW slots is given in [27]. The authors consider a network of IEEE 802.11ah devices in a scenario, when a group of STAs, each having one data frame to transmit, simultaneously start accessing the channel. The authors develop an analytical model, which allows finding the probability that (a) an arbitrarily chosen STA of the group, or (b) all the STA of the group succeed to transmit data by the end of the RAW slot. In contrast to our paper, the model in [27] does not consider the energy consumption of STAs and does not describe the scenario when STAs can drop out of the transmission process due to the depletion of energy.

The model from [27] is extended in [28,49] for scenarios with a random amount of traffic at each STA and anycast transmissions correspondingly.

## 4. Mathematical Model

In this section, we consider the scenario described in Section 2.2. Specifically, we consider a Wi-Fi network where an AP collects data from a group of energy-harvesting sensor STAs, and uses the Periodic RAW in order to organize the STA transmissions. The AP divides the STAs into groups and allocates a series of RAW slots to every group. The AP faces the problem of efficient resource allocation to the STAs: given the number of STAs, the STA traffic intensity, the channel properties and the required probability of data delivery, the AP should find such a way to divide the STAs into groups and to set the RAW slot durations for each group that the probability of data delivery is not less than the required value, but at the same time the amount of occupied channel resources, i.e., the total duration of RAW slots, is minimal.

To solve this problem, we consider an opposite task: given the number of STAs that transmit their data in a single RAW slot and the RAW slot duration, we develop a mathematical model of data transmission that can be used to find the probability of data delivery by an STA. This model can be used in order to find the RAW slot duration that, for a given number of STAs, can guarantee the required probability of data delivery. We use this result in a more general model that describes the data transmission with Periodic RAW on a large scale: given the number of STAs and the number of groups, it can be used to calculate the amount of channel resources consumed by the STAs. We further use the general model to find the best way to divide the STAs into groups and thus solve the problem.

In Section 4.1, we study how the amount of occupied channel time depends on the number of groups. In Section 4.2, we develop a model of the data transmission process in a single RAW slot that allows us to find the minimal duration of the RAW slot that guarantees the given probability of successful frame delivery. The most important symbols used in the model are summarized in Table 1.

### 4.1. Periodic RAW

Let the number of sensors connected to the AP equal $N_{0}$. The AP tries to divide these STAs into *G* groups of the same size. As $N_{0}$ may be not divisible by *G*, we assume that the first $G_{1}=N_{0}\;\mathrm{mod}\;G$ groups contain $N_{1}=\left\lceil \frac{N_{0}}{G}\right\rceil$ STAs, and the rest $G_{2}=G-G_{1}$ groups contain $N_{2}=\left\lfloor \frac{N_{0}}{G}\right\rfloor$ STAs. Then, the AP periodically allocates a series of RAW slots, as shown in Figure 1. The RAW slots do not intersect and can be considered separately. Once in *T* time units, each group obtains a RAW slot.

Let $p_{in}$ be the probability of a new frame arrival to the STA’s transmission queue by the beginning of its RAW slot. As we consider $T=D_{max}$, each STA has no more than one frame in the queue during each RAW slot.

Let us consider a group and randomly choose an STA that has a frame to transmit and further call it *the chosen STA*. For convenience, the remaining STAs from its group are referred to as *other* STAs.

Let the number of STAs in the group equal $N_{g}$, and the RAW slot duration equals $T_{raw}$. The probability that the chosen STA makes a successful transmission in a RAW slot equals

(2) StotalNg,Traw=∑n=0Ng−1Ng−1npinn1−pinNg−1−nSrawn+1,Traw,

where $S_{raw}\left(n+1,T_{raw}\right)$ is the probability that the chosen STA succeeds to transmit its frame in a RAW slot with duration $T_{raw}$ provided that n+1 STAs are trying to send a frame. We multiply this probability by the probability of *n* other STAs to generate a frame by the beginning of the RAW slot and sum over all possible values of *n*. We develop a model to calculate the $S_{raw}\left(n+1,T_{raw}\right)$ in Section 4.2.

To guarantee that the frame is delivered during the RAW slot, the RAW slot duration should be chosen in such a way that the probability of successful transmission equals or is greater than the threshold $p_{req}$. At the same time, it should be as small as possible and equals

(3) $$T_{min}\left(N_{g}\right)=\min_{T_{raw}}\left\{T_{raw}:S_{total}\left(N_{g},T_{raw}\right)\geq p_{req}\right\}.$$

We have two possible group sizes, $N_{1}$ and $N_{2}$, which yield different optimal RAW slot durations. Our goal is to find the optimal number of groups that yields the minimal amount of occupied channel resources, which is calculated as

(4) $$\begin{array}{cc} G_{opt} & =\arg\min_{G}\frac{T_{min}\left(N_{1}\right)\times G_{1}+T_{min}\left(N_{2}\right)\times G_{2}}{T}\\ \; & =\arg\min_{G}\frac{T_{min}\left(\lceil \frac{N_{0}}{G}\rceil \right)\times \left(N_{0}\;\mathrm{mod}\;G\right)+T_{min}\left(\lfloor \frac{N_{0}}{G}\rfloor \right)\times \left(G-N_{0}\;\mathrm{mod}\;G\right)}{T}. \end{array}$$

To find the $G_{opt}$, we can use the exhaustive search over $G\in \left[1,N_{0}\right]$, but we need to know the distribution $S_{raw}\left(n+1,T_{raw}\right)$ that is obtained as described in the next subsection.

### 4.2. RAW Slot

Let us consider a RAW Slot, during which *N* STAs try to send their frames according to EDCA channel access. Since there are no hidden STAs in the network, the STAs decrement their backoff counters synchronously. Following [48,50], we refer to a time interval between two consequent changes of the backoff counter as a *virtual slot*.

A virtual slot can be:

- *empty* if none of the STAs transmits a frame;
- *successful* if only one STA accesses the channel, and the frame transmission is not affected by the noise;
- *unsuccessful* if only one STA accesses the channel, but the frame is damaged by the noise;
- *collision* if more than one STAs access the channel.

In the model, we consider that the empty slot duration is σ, while the duration of other slots is τ. The non-empty virtual slot duration is calculated as $\tau =SIFS+D_{dat}+D_{ack}+AIFS$, where $D_{dat}$ is the data frame duration and $D_{ack}$ is the acknowledgement duration.

Let us enumerate all virtual slots in the RAW slot starting from zero and observe the data transmission process at the beginning of each virtual slot. We describe the state of the system in the virtual slot *t* as $\eta_{t}=\left(n,f,r\right)$, where *n* is the number of active STAs, i.e., those STAs that have both data and energy to transmit, *f* is the number of passed non-empty virtual slots, and *r* is the value of the chosen STA’s retry counter. Thus, we define a discrete-time Markov chain with the time unit equal to a virtual slot and the state $\eta_{t}=\left(n,f,r\right)$, where 1≤n(t)≤N, 0≤f(t)≤t, 0≤r(t)≤RL−1. The process can also transit to the absorbing state denoted as ∗ when at least one of the event occurs:

- the chosen STA transmits its frame successfully, or
- it runs out of energy, or
- its retry counter reaches RL, or
- the RAW slot is terminated.

Note that *n* includes only the STAs having enough energy to transmit their frames and having undelivered frames in the queue with less than RL transmission attempts.

The initial state of the process is (N,0,0), where *N* is the number of active STAs in the group.

Let $T_{real}\left(t,f\right)$ be the real time interval from the beginning of the RAW to the beginning of the slot number *t*, provided that *f* previous slots were not empty:

(5) $$T_{real}\left(t,f\right)=f\tau +\left(t-f\right)\sigma .$$

Let the traffic form a Poisson flow. Then, the distribution of the time elapsed from the last transmission attempt is exponential. Thus, by the beginning of the RAW slot, the STA has a random amount *Q* of energy, distributed exponentially:

(6) $$P\left(Q\leq x\right)=1-e^{-\frac{x}{\langle Q\rangle }},$$

where 〈Q〉 is the average value of energy. Such an energy distribution is similar to the one considered in [34,51].

Let the STA consume the Δq amount of energy in a virtual slot. The remaining energy of an STA after this virtual slot, provided that it has had enough energy to survive until the end of this slot has an inverse CDF, equal to Pr(Q>q+Δq|Q≥Δq). According to the memory-less property of the exponential distribution, it equals the initial inverse CDF:

(7) Pr(Q>q+Δq|Q≥Δq)=Pr(Q>q),

i.e., the distribution of the STA’s energy at the beginning of each virtual slot is the same, provided that it has had enough energy to stay alive. In the model, we neglect the amount of energy harvested by the STA during its RAW slot because the STA transmits only one frame, and we consider that the transmission time is negligible in comparison with the RAW period.

**Figure 2:**
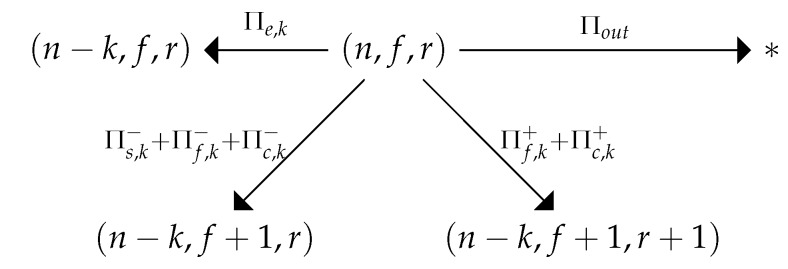
The possible transitions when the retry limit is not reached.

Let us describe the possible transitions from the state $\eta_{t}$. We use the parameter *k* to indicate the number of *other* STAs that turn off their radio by the end of the slot *t* (because of the lack of energy or because of the successful transmission). Symbol “+” indicates the transition, in which the chosen STA transmits a frame. Otherwise, symbol “−” is used. The transitions described below are shown in Figure 2. These transitions correspond to a case when the STA’s retry counter has not reached the RL.

- With probability $\Pi_{out}\left(\eta_{t}\right)$, the process transits to the absorbing state $\eta_{t+1}=*$.
- With probability $\Pi_{e,k}\left(\eta_{t}\right)$, the slot is empty and *k* other STAs run out of energy. Thus, the process transits to $\eta_{t+1}=\left(n-k,f,r\right)$.
- With probability $\Pi_{s,k}^{-}\left(\eta_{t}\right)$, the slot is successful, the chosen STA does not try to transmit, and k−1 other STAs run out of energy; the process transits to $\eta_{t+1}=\left(n-k,f+1,r\right)$.
- With probability $\Pi_{f,k}^{-}\left(\eta_{t}\right)$, the slot is unsuccessful, the chosen STA does not try to transmit, and *k* other STAs run out of energy. The process transits to $\eta_{t+1}=\left(n-k,f+1,r\right)$.
- With probability $\Pi_{c,k}^{-}\left(\eta_{t}\right)$, the slot is collision, the chosen STA does not try to transmit, and *k* other STAs run out of energy. The process transits to $\eta_{t+1}=\left(n-k,f+1,r\right)$.
- With probability $\Pi_{f,k}^{+}\left(\eta_{t}\right)$, the chosen STA tries to transmit a frame, the slot is unsuccessful, and *k* other STAs run out of energy. The process transits to $\eta_{t+1}=\left(n-k,f+1,r+1\right)$.
- With probability $\Pi_{c,k}^{+}\left(\eta_{t}\right)$, the slot is collision, the chosen STA tries to transmit a frame, and *k* other STAs run out of energy. The process transits to $\eta_{t+1}=\left(n-k,f+1,r+1\right)$.

**Figure 3:**
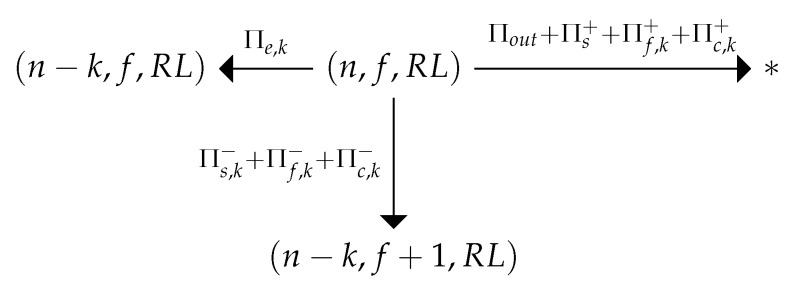
The possible transitions when the retry limit is reached.

If the STA’s retry counter *r* reaches RL, then the process transitions corresponding to the cases when the chosen STA tries to transmit, but the slot is unsuccessful (with probability $\Pi_{f,k}^{+}\left(\eta_{t}\right)$) or collision (with probability $\Pi_{c,k}^{+}\left(\eta_{t}\right)$) also leads to the absorbing state $\eta_{t+1}=*$, as shown in Figure 3.

We need some auxiliary values to present the transition probabilities.

#### 4.2.1. The Probability of Chosen STA Transmitting a Frame

We need to find the probability Pr(TX|t,r) of the chosen STA to transmit a frame, provided that the slot number equals *t*, and the STA has made *r* transmission attempts. Let us define the following probabilities:

- Pr(t,r) is the probability of the considered process not to transit to the absorbing state and the chosen STA making *r* transmission attempts by the moment *t*.
- Pr(A,t,r) is the probability of the considered process by the moment *t* not to transit to the absorbing state, the chosen STA making *r* transmission attempts and an event $A\in \left\{TX,C,S\right\}$ occurring:
  – TX means that the STA makes a transmission attempt.
  – *C* means that the STA makes a transmission attempt that leads to collision.
  – *S* means that the STA makes a transmission attempt that does not lead to collision.

It is obvious that Pr(TX,t,r)=Pr(C,t,r)+Pr(S,t,r).

By definition, we have:

(8) $$\mathrm{Pr}\left(TX|t,r\right)=\frac{\mathrm{Pr}(TX,t,r)}{\mathrm{Pr}(t,r)}.$$

The numerator can be written as follows:

(9) $$\mathrm{Pr}\left(TX,t,r\right)=\left\{\begin{array}{cc} \frac{1}{CW_{0}}, & r=0,0\leq t<CW_{0},\\ 0, & r=0,t\geq CW_{0},\\ 0, & r\geq RL,\\ \sum_{i=t-CW_{r}}^{t-1}\frac{\mathrm{Pr}(C,i,r-1)+p\mathrm{Pr}(S,i,r-1)}{CW_{r}}, & 0<r<RL. \end{array}\right.$$

The first three equalities are obvious. The last one corresponds to transmission retries. After a collision or a frame being damaged by the noise, the STA chooses one of $CW_{r}$ virtual slots, each with probability $\frac{1}{CW_{r}}$. The probability of transmission being disrupted by the noise equals pPr(S,t,r). Then, the probability of collision or of the frame being damaged by noise equals Pr(C,t,r)+pPr(S,t,r).

Let us find Pr(t,r). Note that the retry-counter equals *r* at the beginning of slot *t* if and only if it has turned *r* in one of the previous slots, and, since that moment, the chosen STA has not tried to transmit. In this case:

(10) $$\mathrm{Pr}\left(t,r\right)=\left\{\begin{array}{cc} 1-\sum_{i=0}^{t-1}\mathrm{Pr}\left(TX,i,r\right), & r=0,\\ \sum_{i=0}^{t-1}\mathrm{Pr}\left(C,i,r-1\right)+p\sum_{i=0}^{t-1}\mathrm{Pr}\left(S,i,r-1\right)-\sum_{t=0}^{t-1}\mathrm{Pr}\left(TX,i,r\right), & r>0. \end{array}\right.$$

For an infinite number of STAs, the transmission probability equals the probability of collision: Pr(TX,t,r)=Pr(C,t,r). Let us define values a(t,r) and b(t,r), in which Pr(TX,t,r) and Pr(t,r) turn when the number of STAs is infinite. These values are determined by the following formulae:

(11) $$a\left(t,r\right)=\left\{\begin{array}{cc} \frac{1}{CW_{0}}, & r=0,0\leq t<CW_{0},\\ 0, & r=0,t\geq CW_{0},\\ 0, & r\geq RL,\\ \sum_{i=t-CW_{r}}^{t-1}\frac{a(i,r-1)}{CW_{r}}, & 0<r<RL, \end{array}\right.$$

(12) $$b\left(t,r\right)=\left\{\begin{array}{cc} 1-\sum_{i=0}^{t-1}b\left(i,r\right), & r=0,\\ \sum_{i=0}^{t-1}b\left(i,r-1\right)-\sum_{t=0}^{t-1}b\left(i,r\right), & r>0. \end{array}\right.$$

Instead of Pr(TX|t,r), we use its approximation $u\left(t,r\right)=\frac{a(t,r)}{b(t,r)}$, corresponding to the case, when the transmission probability equals the collision probability. In [27], it is shown that the error induced by such approximation is negligibly small even for N=10 STAs and decreases with the growth of *N*.

#### 4.2.2. The Probability of Other STA Transmitting a Frame

Let us randomly choose an STA from those n−1 other STAs that are still alive by the slot *t*. Let us find the probability $\mathrm{Pr}(TX|t,\hat{n},\hat{f})$ of this STA making a transmission attempt in instant *t* with given $n=\hat{n}$ and $f=\hat{f}$. By definition

(13) $$\mathrm{Pr}\left(TX|t,\hat{n},\hat{f}\right)=\frac{\sum_{\hat{r}}\mathrm{Pr}\left(TX,\eta_{t}=\left(\hat{n},\hat{f},\hat{r}\right)\right)}{\sum_{\hat{r}}\mathrm{Pr}\left(\eta_{t}=\left(\hat{n},\hat{f},\hat{r}\right)\right)},$$

where $\mathrm{Pr}(\eta_{t})$ is the probability of the considered process to be found in state $\eta_{t}$ at the moment *t*, and $\mathrm{Pr}(TX,\eta_{t})$ is the probability of considered process to be found in state $\eta_{t}$ at the moment *t* and the randomly chosen STA making a transmission attempt.

By the law of total probability,

(14) $$\mathrm{Pr}\left(TX,\eta_{t}\right)=\mathrm{Pr}\left(TX|\eta_{t}\right)\mathrm{Pr}\left(\eta_{t}\right).$$

It is obvious that the probability of an STA transmitting in slot *t* depends only on the slot number and on the number of its transmission attempts, i.e., $\mathrm{Pr}\left(TX|\eta_{t}=\left(\hat{n},\hat{f},\hat{r}\right)\right)=\mathrm{Pr}\left(TX|t,\hat{r}\right)$. Let us replace Pr(TX|t,r) with its approximate value u(t,r) and introduce a value

(15) $$v\left(t,\hat{n},\hat{f}\right)=\frac{\sum_{\hat{r}=0}^{\min(f,RL-1)}u\left(t,\hat{r}\right)\mathrm{Pr}\left(\eta_{t}=\left(\hat{n},\hat{f},\hat{r}\right)\right)}{\sum_{\hat{r}=0}^{\min(f,RL-1)}\mathrm{Pr}\left(\eta_{t}=\left(\hat{n},\hat{f},\hat{r}\right)\right)},$$

which approximates $\mathrm{Pr}(TX|t,\hat{n},\hat{f})$ and coincides with it in case of the infinite number of STAs. The limits of summation over $\hat{r}$ are explained by the fact that the number of retries does not exceed the number of non-empty slots and does not exceed the retry limit.

#### 4.2.3. Additional Values

Let us introduce some more auxiliary values that determine the transmission probabilities in the state $\eta_{t}$:

- $\pi_{k}\left(\eta_{t}\right)$ is the probability of *k* other STAs transmitting in slot *t*,
- $\pi_{e}\left(\eta_{t}\right)$ is the probability of none of the STAs transmitting in slot *t*,
- $\pi_{s}^{+}\left(\eta_{t}\right)$ is the probability of only the chosen STA transmitting in slot *t*,
- $\pi_{s}^{-}\left(\eta_{t}\right)$ is the probability of one other STA transmitting in slot *t*,
- $\pi_{c}^{+}\left(\eta_{t}\right)$ is the probability of the chosen STA and at least one other STA transmitting in slot *t*,
- $\pi_{c}^{-}\left(\eta_{t}\right)$ is the probability of the chosen STA not transmitting in slot *t*, but at least two other STAs transmitting in this slot.

These values by definition equal zero when $\eta_{t}=*$. Let us express these values in terms of u(t,r) and v(n,t,f):

(16) πkηt=(n,f,r)=n−1kv(t,n,f)k1−v(t,n,f)n−k−1,πeηt=(n,f,r)=1−u(t,r)π0(ηt),πs+ηt=(n,f,r)=u(t,r)π0(ηt),πs−ηt=(n,f,r)=1−u(t,r)π1(ηt),πc+ηt=(n,f,r)=u(t,r)(1−π0(ηt)),πc−ηt=(n,f,r)=1−u(t,r)1−π0(ηt)−π1(ηt).

For the purpose of brevity, we hereinafter omit the argument $\eta_{t}=\left(n,f,r\right)$ for probabilities of transmission π and transition Π.

#### 4.2.4. The Probability of the Process to Transit to the Absorbing State

We assume that the STA spends $q_{e}$ energy units in an empty slot, $q_{rs}$ energy units in a successful slot if it does not transmit and $q_{ts}$ energy units if it transmits, $q_{rf}$ energy units in an unsuccessful or collision slot if it does not transmit and $q_{tf}$ energy units if it transmits. The energy consumption values are calculated as follows:

(17) $$\begin{array}{cc} q_{e} & =V\sigma I_{LS},\\ q_{rf} & =V\left(D_{dat}I_{RX}+\left(SIFS+D_{Ack}+AIFS\right)I_{LS}\right),\\ q_{rs} & =V\left(\left(D_{dat}+D_{Ack}\right)I_{RX}+\left(SIFS+AIFS\right)I_{LS}\right),\\ q_{tf} & =V\left(D_{dat}I_{TX}+\left(SIFS+D_{Ack}+AIFS\right)I_{LS}\right),\\ q_{ts} & =V\left(D_{dat}I_{TX}+D_{Ack}I_{RX}+\left(SIFS+AIFS\right)I_{LS}\right), \end{array}$$

where *V* is the voltage and $I_{LS}$, $I_{RX}$, $I_{TX}$ are the current values, consumed by the STA while listening to the channel, receiving and transmitting, respectively.

The way the probability of transition to the absorbing state is calculated depends on the current state:

- If the STA cannot transmit the frame by the end of the RAW, i.e., $T_{raw}-T_{real}\left(t,f\right)<\tau$, the process transits to the absorbing state, i.e., $\Pi_{out}=1$.
- Otherwise, two situations are possible:
  – if r≠RL−1, the process transits to the absorbing state if the frame transmission is successful, or if the chosen STA runs out of energy:
    (18) $$\begin{array}{cc} \Pi_{out} & =\mathrm{Pr}\left(Q<q_{e}\right)\pi_{e}+\mathrm{Pr}\left(Q<q_{ts}\right)p\pi_{s}^{+}\\ \; & +\mathrm{Pr}\left(Q<q_{tf}\right)\pi_{c}^{+}+\mathrm{Pr}\left(Q<q_{rs}\right)\pi_{s}^{-}\\ \; & +\mathrm{Pr}\left(Q<q_{rf}\right)\pi_{c}^{-}+\left(1-p\right)\pi_{s}^{+}. \end{array}$$
  – if r=RL−1, the process transits to the absorbing state on any transmission attempt, or if the chosen STA runs out of energy:
    (19) $$\Pi_{out}=\mathrm{Pr}\left(Q<q_{e}\right)\pi_{e}+\mathrm{Pr}\left(Q<q_{rs}\right)\pi_{s}^{-}+\mathrm{Pr}\left(Q<q_{rf}\right)\pi_{c}^{-}+\pi_{s}^{+}+\pi_{c}^{+}.$$

Note that all other transitions take place only if $T_{raw}-T_{real}\left(t,f\right)\geq \tau$.

#### 4.2.5. The Probability of the Slot Being Empty

To find $\Pi_{e,k}$ we take into account that none of the STAs access the channel and *k* other STAs run out of energy:

(20) Πe,k=πen−1kPr(Q<qe)kPr(Q≥qe)n−k.

#### 4.2.6. The Probability of the Slot Being Successful and the Chosen STA Not Trying to Transmit

After a successful transmission, the transmitting STA turns of, therefore k−1 more STAs, must run out of energy to make a total of *k* STAs that turn off by the end of the slot:

(21) Πs,k−=(1−p)πs−n−2k−1Pr(Q<qrs)k−1Pr(Q≥qrs)n−k.

#### 4.2.7. The Probability of the Slot Being Unsuccessful and the Chosen STA Not Trying to Transmit

If the transmitting STA has enough energy to transmit in the following slot, *k* STAs must run out of energy. Otherwise, only k−1 STAs must run out of energy:

(22) Πf,k−=pπs−(n−2kPr(Q≥qtf)Pr(Q<qrf)kPr(Q≥qrf)n−k−1+n−2k−1Pr(Q<qtf)Pr(Q<qrf)k−1Pr(Q≥qrf)n−k).

#### 4.2.8. The Probability of the Slot Being Collision and the Chosen STA Not Trying to Transmit

In the given formula, we sum over the number of STAs *i* that participate in the collision. For each *i*, we go over the number of STAs *j* that participate in the collision and run out of energy. If *j* STAs participating in the collision run out of energy, then k−j STAs not participating in the collision must run out of energy too, i.e.,

(23) Πc,k−=(1−v(n,r))Pr(Q≥qrf)∑i=2n−1{πi∑j=0min(i,k)[ijn−1−ik−j×Pr(Q<qtf)jPr(Q≥qtf)i−jPr(Q<qrf)k−jPr(Q≥qrf)n−i−k+j−1]}.

#### 4.2.9. The Probability of the Chosen STA Trying to Transmit and the Slot Being Unsuccessful

This event takes place when the chosen STA alone gains access to the channel, its transmission is disrupted by the noise, and it has already made r≠RL−1 transmission attempts. In addition, *k* other STAs have to run out of energy. The probability of this event equals:

(24) Πf,k+=pπs+n−1kPr(Q≥qtf)Pr(Q<qrf)kPr(Q≥qrf)n−k−1.

#### 4.2.10. The Probability of the Chosen STA Trying to Transmit and the Slot Being Collision

This transition takes place if r≠RL−1. As in the case when the chosen STA does not transmit, we sum over the number *i* of STAs, taking part in the collision. The difference is that the lower limit of *i* is 1:

(25) Πc,k+=v(n,r)Pr(Q≥qtf)∑i=1n−1{πi∑j=0min(i,k)[ijn−1−ik−j×Pr(Q<qtf)jPr(Q≥qtf)i−jPr(Q<qrf)k−jPr(Q≥qrf)n−i−k+j−1]}.

### 4.3. The Probability of the Chosen STA Transmitting Its Frame Successfully

The considered process in initially in state $\eta_{0}=\left(N,0,0\right)$, where *N* is the initial number of STAs:

(26) $$\mathrm{Pr}\left(\eta_{0}=\left(n,f,r\right)\right)=\left\{\begin{array}{cc} 1 & n=N,f=0,r=0,\\ 0 & \mathrm{otherwise}. \end{array}\right.$$

The possible transitions from state $\eta_{t}=\left(n,f,r\right)$ are shown in Figure 2 and Figure 3. Let us know the distribution $\mathrm{Pr}(\eta_{t})$ for the moment *t*. Then, we can use the transition probabilities Π to calculate the distribution $\mathrm{Pr}(\eta_{t+1})$ for the next time moment.

The probability of the chosen STA transmitting its frame successfully during the RAW is calculated as:

(27) $$S_{raw}\left(N,T_{raw}\right)=\sum_{t,\eta_{t}}\mathrm{Pr}\left(\eta_{t}\right)\left(1-p\right)\pi_{s}^{+}\left(\eta_{t}\right).$$

It is sufficient to sum over those *t*, for which $T_{raw}-T_{real}\left(t,f\right)<\tau$ because $\pi_{s}^{+}\left(*\right)=0$. Thus, to find $S_{raw}$, we consider the evolution of the transmission process starting with its initial state at time t=0, and iteratively calculate the probability of the process to reach its possible states at time *t* until either the real time exceeds $T_{raw}-\tau$, or the total probability of the process to reach an absorbing state becomes close to 1. The calculations can be hastened by omitting the states with low (in comparison with the required accuracy) probability. We further use $S_{raw}$ in Equation (2) to obtain the probability of successful transmission by the chosen STA in the RAW slot and further to find the optimal number of groups.

## 5. Numerical Results

In this section, we present and analyze the numerical results obtained using the designed models. In Section 5.1, we consider a single RAW slot and examine the dependence of the probability of successful transmission on different parameters, such as the average STA energy, probability of transmission being disrupted by noise, and the number of STAs. In Section 5.2, we consider the Periodic RAW and show the dependency of the amount of allocated channel resource on the number of groups.

### 5.1. RAW Slot

We assume that the STAs operate in a 2 MHz channel and use the most reliable modulation coding scheme (MCS0). The STAs transmit 100-byte data frames. Table 2 lists the experiment parameters. Specifically, the values of channel access parameters are given in the IEEE 802.11ah [10] amendment. Voltage *V* and the values of current $I_{LS}$, $I_{RX}$, $I_{TX}$, consumed by the STA while listening to the channel, receiving, and transmitting, respectively, are given in the IEEE simulation scenario recommendations [52].

**Figure 4:**
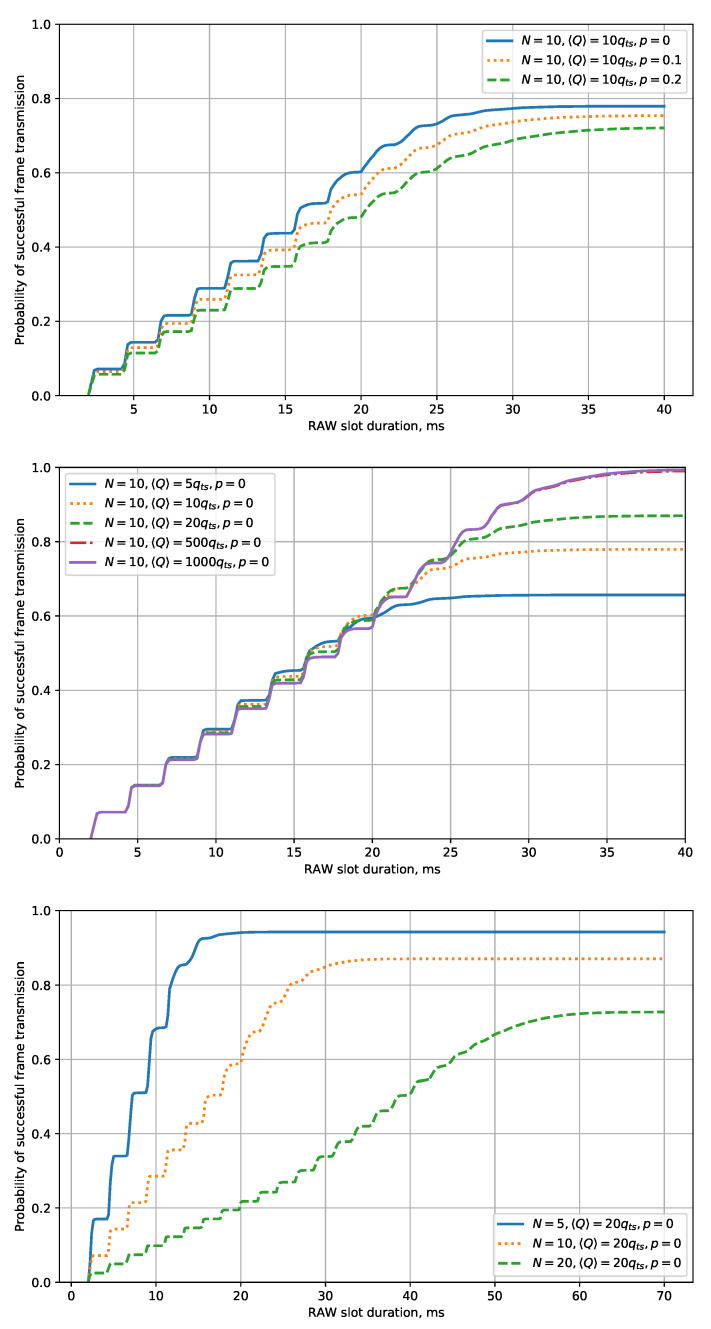
The dependency of the probability of successful frame transmission on the RAW slot duration for various *p*, 〈Q〉, and *N*.

Figure 4 shows the dependency of the probability of successful frame transmission during the RAW slot on the RAW slot duration for various values of N,〈Q〉,p. The shown dependencies are monotonic, and the probability of success asymptotically approaches some value. This value is less than one because the STA’s energy is limited. In addition, this value decreases with an increase of *N* and *p* and with a decrease of 〈Q〉. This result is expected because the greater initial number of STAs yields the greater probability of their frames to enter a collision, and retries, caused by the collisions or noise, result in higher energy consumption. As a result, the STA can deplete its energy before successful transmission.

Before the probability becomes constant, it grows “step-by-step” with the increase of the RAW slot duration. Such behavior is directly related to the fact that, with the increase of the RAW slot duration, the number of frames that can be transmitted within the RAW slot increases in a step-type manner. The width of a “step” is determined by the non-empty slot duration of τ and approximately equals $D_{dat}+SIFS+D_{Ack}+AIFS$. The height of a “step” is determined by the number of STAs *N* and the initial contention window $CW_{0}$. To find it, let us consider the beginning of the RAW slot and assume that all the STAs have sufficient energy to make the first transmission attempt. The first step of the plot corresponds to a situation when the chosen STA makes a successful first transmission attempt, and other STAs do not transmit their frames before the chosen STA. For slot number *k*, the probability of such an event equals

(28) $$\frac{1}{CW_{0}}{\left(\frac{CW_{0}-k-1}{CW_{0}}\right)}^{N-1},$$

where the first multiplier is the probability of the chosen STA selecting the considered slot for transmission, and the expression in brackets is the probability that the other STAs select slots after the considered one. Summing over all *k* possible for the first transmission attempt, we obtain the step height:

(29) $$H=\frac{\sum_{k=1}^{CW_{0}}k^{N-1}}{CW_{0}^{N}}.$$

The steps become more and more smooth with the increase of the RAW slot duration.

Let us find the minimal $T_{raw}$, which yields the required probability $p_{req}$ of successful transmission. This value may not exist if the average STA’s energy is too low, or there are too many STAs, or the noise level is too high. For example, in case N=10, $\langle Q\rangle =20q_{ts}$, p=0, the probability of successful transmission of 0.9 cannot be achieved with any duration of RAW slot. However, with $\langle Q\rangle =500q_{ts}$ and $\langle Q\rangle =1000q_{ts}$, the required probability is reached with $T_{raw}\approx 28$ ms. Such a result shows the importance of the developed model: the models known from the literature, e.g., [27,49], do not consider the STA energy and would provide results similar to the results with a high 〈Q〉. Thus, unlike the models known from the literature, the developed model shows us that, in some cases, we cannot achieve high probabilities of successful data transmission by increasing the RAW slot duration.

At the same time, the developed model gives us possible solutions for the considered case. The first one, if the considered devices gain energy from a renewable source (e.g., from a solar battery), is to delay the beginning of the RAW slot to let the STAs accumulate more energy, thus increasing 〈Q〉. Another solution is to divide the STAs into two groups and thus to decrease the parameter *N* from 10 to 5. In such a case, the required probability of successful transmission becomes achievable with $T_{raw}\approx 15$ ms.

The grouping approach might be more efficient because the growth of the probability of successful transmission is not directly proportional to the number of STAs (see Figure 4).

### 5.2. Periodic RAW

**Figure 5:**
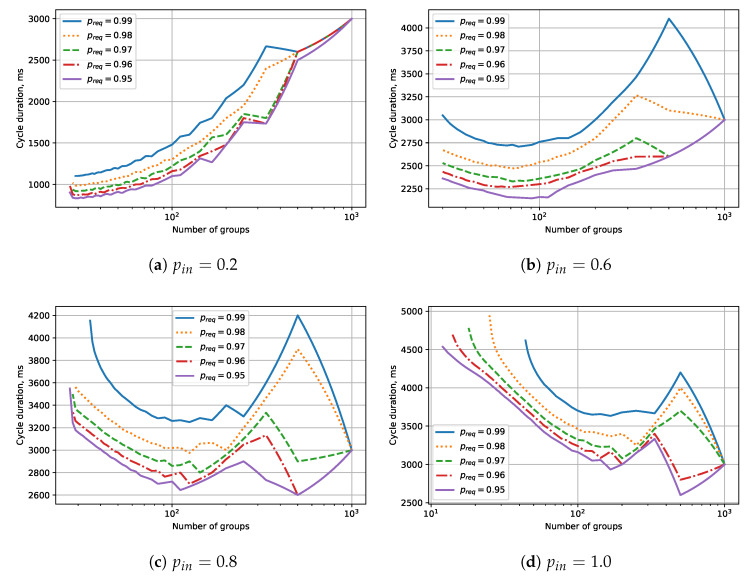
The dependency of the cycle duration on the number of groups for different data generation probability $p_{in}$.

Let us now investigate the case with periodic RAW. As a performance indicator of the network, we consider the cycle duration, which is the total duration of RAW slots (the numerator of the fraction in Equation (4)) within a single RAW period. The numerical results obtained for $N_{0}=1000$ battery-powered energy harvesting STAs and $\langle Q\rangle =1000q_{ts}$ are shown in Figure 5. We consider the $p_{req}$ values close to 100% within a small interval starting with 95%. The dependency of the cycle duration on the number of groups is non-monotonic, with extremum points corresponding to such numbers of groups by which the number of STAs is divisible. As one can see, a minimum minimorum exists that depends on the required probability of successful transmission $p_{req}$.

The curves corresponding to different $p_{req}$ values converge when the number of groups equals the number of STAs. In this extreme case, every STA obtains a RAW slot, the length of which includes the time needed by the STA to count down its backoff and to transmit the frame. This length is the same for the considered $p_{req}$ values, and therefore the cycle durations are the same.

The important point is that small numbers of groups (and big numbers of STAs in a group) make the desired probability of success unachievable for any value of $T_{raw}$ because STAs deplete their energy. Another point worth mentioning is that the cycle duration significantly depends on $p_{req}$. Even though the considered $p_{req}$ values are close to 100%, even a 1% difference can bring a significant difference in the cycle duration. Specifically, with the increase of $p_{req}$, it can suddenly change from increasing to decreasing dependency. This peculiarity is caused by the “step-by-step” dependency of the probability of successful transmission on the RAW duration.

**Figure 6:**
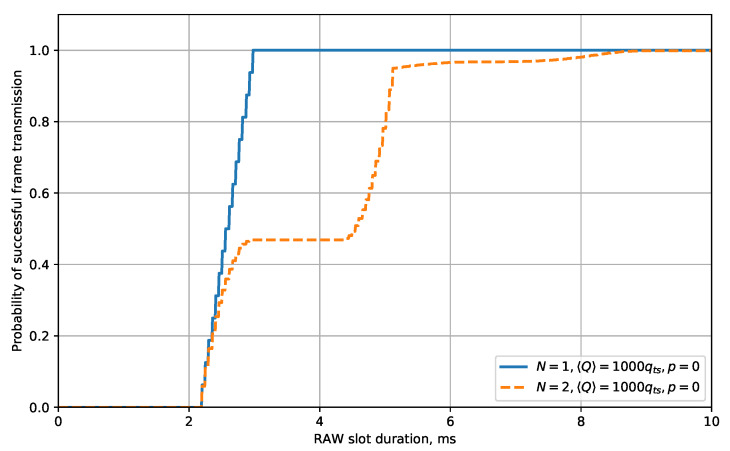
The dependency of the probability of successful frame transmission on the RAW duration for small numbers of STAs.

To explain this fact, let us consider a case with $p_{in}=1$ and $N_{0}=1000$ (see Figure 5d). If we divide STAs into 1000 groups, each group will contain exactly 1 STA. The duration of the RAW that each group obtains in the general case is determined by $p_{req}$, but, for 1 STA, it equals 2.98 for all considered $p_{req}$ values (see Figure 6). If we divide STAs into 500 groups, each group will contain exactly 2 STAs. In this case, the duration of the RAW slot varies significantly for different $p_{req}$ values. For $p_{req}=0.95$, minimal RAW slot duration equals 5.18 ms, so if we exchange two 1-STA groups for one 2-STA group, we win 0.78 ms. However, for $p_{req}=0.99$, minimal RAW slot duration equals 8.36 ms, so, if we exchange two 1-STA groups for one 2-STA group, we lose 2.42 ms.

## 6. Conclusions

Wi-Fi HaLow aims at extending the usage of Wi-Fi to the Internet of Things scenarios, which include gathering data from large numbers of autonomous energy-limited devices. An efficient tool that can help to reach this goal is the novel mechanism, introduced in the IEEE 802.11ah standard amendment: the Restricted Access Window (RAW). The Wi-Fi Access Point can use RAW to divide the connected STAs into groups and assign them a series of time intervals—RAW slots—during which only the STAs from a given group can transmit their data, thus decreasing the contention for channel access.

In the paper, we have studied an extreme scenario in which the STAs are energy-harvesting sensors that harvest energy with time and consume it during the data transmission, and it is possible that during the process of channel access and data transmission, the devices run out of energy. For such a scenario, we have developed an analytical model of data transmission that can be used to find the probability of successful data transmission by an STA as a function of the number of STAs, average STA energy, RAW slot duration, and the probability of a transmission to be damaged by random noise. Such a model can be used to determine the minimal RAW duration that, for a given number of STAs, can guarantee the given probability of data delivery. Unlike the existing models that do not take into account the energy consumption of devices, the developed model shows that, in some cases, the required probability of data delivery cannot be achieved by increasing the RAW slot duration. We have used this model to solve the STA grouping problem: how to divide the STAs into groups in such a way that all the STAs can transmit their data with required probability, and the portion of consumed channel resources is minimal. While solving the STA grouping problem, we have obtained and explained some paradoxical results, e.g., increasing the number of groups and correspondingly decreasing the number of STAs per group does not necessarily provide lower channel resource consumption. At the same time, the model can be used to find the optimal number of groups and the corresponding duration of RAW slots for each group which can decrease the cycle duration by almost 50% in comparison with the usage of a single group for all devices or with the usage of a group for each device.

Let us summarize the contributions of the paper:

- We have developed a model of data transmission in the RAW slot when STAs transmit single packets, the STAs have limited amounts of energy, and their transmissions can be disrupted by random noise. The model allows us to calculate the probability that the data are delivered with a given RAW configuration.
- We have shown that it is important to consider the amount of STAs’ energy in order to properly configure the RAW parameters, while the usage of models that do not consider the STA energy consumption may violate the requirements on the reliability of communications.
- We have shown how to use the developed model to optimize the RAW slot duration in order to provide the required probability of data delivery and to minimize the amount of consumed channel resources.
- We have shown that the channel resource consumption can change drastically depending on the reliability requirements: changing the required probability by 1% can increase the consumption by almost 100%, so the RAW parameters optimization shall take into account the requirements of particular applications.

As a direction of future work, we consider the modeling of data transmission for more complex models of traffic and energy harvesting. We also plan to consider the usage of RAW in heterogeneous networks. 

## Figures and Tables

**Table 1:** Model notation.

Symbol	Meaning
$N_{0}$	Total number of STAs in the network
*G*	Number of STA groups
$N_{1},N_{2}$	Number of STAs in big and small groups
$G_{1},G_{2}$	Number of big and small groups
*T*	RAW period
$D_{max}$	Maximal transmission delay
$p_{in}$	Probability of an STA having data to transmit in its RAW slot
$T_{raw}$	RAW slot duration
$S_{raw}\left(n,T_{raw}\right)$	Probability of the STA making a successful transmission in a RAW slot to which *n* STAs are assigned
$S_{total}$	Probability of the STA making a successful transmission during a RAW period
σ	Empty slot duration
τ	Non-empty slot duration
$D_{dat}$	Data frame duration
$D_{ack}$	Ack frame duration
*t*	Model time measured in virtual slots from the beginning of the considered RAW slot
$\eta_{t}=\left(n,f,r\right)$	State of the Markov process in virtual slot *t* that describes the transmission within a RAW slot, here *n* is the number of still active STAs, *f* is the number of elapsed non-empty slots, and *r* is the retry counter of the considered STA
RL	Retry limit
$T_{real}\left(t,f\right)$	Real (not virtual) time which corresponds to *t* virtual slots out of which *f* are non-empty
*Q*	Amount of energy which the STA has at the beginning of the RAW slot
*k*	Number of STAs that run out of energy during a virtual slot
$\Pi_{out}$	Probability that the process transits to the absorbing state
$\Pi_{e,k}$	Probability of an empty slot
$\Pi_{s,k}^{+},\Pi_{s,k}^{-}$	Probability of a slot with successful transmission made by the considered STA (+) and by an other STA (-)
$\Pi_{f,k}^{+},\Pi_{f,k}^{-}$	Probability of a slot with unsuccessful transmission made by the considered STA (+) and by an other STA (-)
$\Pi_{c,k}^{+},\Pi_{c,k}^{-}$	Probability of a collision slot involving (+) and not involving (-) the considered STA
$\pi_{k}\left(\eta_{t}\right)$	Probability of *k* other STAs transmitting in slot *t*,
$\pi_{e}\left(\eta_{t}\right)$	Probability of none of the STAs transmitting in slot *t*,
$\pi_{s}^{+}\left(\eta_{t}\right)$	Probability of only the chosen STA transmitting in slot *t*,
$\pi_{s}^{-}\left(\eta_{t}\right)$	Probability of one other STA transmitting in slot *t*,
$\pi_{c}^{+}\left(\eta_{t}\right)$	Probability of the chosen STA and at least one other STA transmitting in slot *t*
$\pi_{c}^{-}\left(\eta_{t}\right)$	Probability of the chosen STA not transmitting in slot *t*, but a collision to happen
*V*	Consumed voltage
$I_{LS},I_{RX},I_{TX}$	Current consumed by the STA for channel listening, receiving and transmitting
$q_{e}$	Energy consumed by the STA during an empty slot
$q_{rf}$	Energy consumed by the STA during a non-empty slot, in which it does not transmit
$q_{rs}$	Energy consumed by the STA during a successful slot, in which it does not transmit
$q_{tf}$	Energy consumed by the STA during a non-empty slot, in which it transmits
$q_{ts}$	Energy consumed by the STA during a successful slot, in which it transmits

**Table 2:** Experiment parameters.

Parameter	Value	Parameter	Value	Parameter	Value
σ	52 μs	τ	2196 μs	$q_{e}$	3 μJ
$D_{Ack}$	240 μs	*V*	1.1 V	$q_{rf}$	202 μJ
$D_{dat}$	1480 μs	$I_{LS}$	50 mA	$q_{rs}$	215 μJ
SIFS	160 μs	$I_{TX}$	280 mA	$q_{tf}$	495 μJ
AIFS	316 μs	$I_{RX}$	100 mA	$q_{ts}$	508 μJ
$CW_{0}$	16	RL	7		

## Abbreviations

The following abbreviations are used in this manuscript:

ACK	Acknowledgement Frame
AIFS	Arbitration Inter-Frame Space
AP	Access Point
CSMA/CA	Carrier Sense Multiple Access with Collision Avoidance
EDCA	Enhanced Distributed Channel Access
IoT	Internet of Things
RAW	Restricted Access Window
SIFS	Short Inter-Frame Space
STA	Station
